# In search of perfect reads

**DOI:** 10.1186/1471-2105-16-S17-S7

**Published:** 2015-12-07

**Authors:** Soumitra Pal, Srinivas Aluru

**Affiliations:** 1 Department of Computer Science and Engineering, Indian Institute of Technology Bombay, Powai, 400076 Mumbai, India; 2 School of Computational Science and Engineering, College of Computing, Georgia Institute of Technology, 266 Ferst Drive, 30332 Atlanta, GA, USA

**Keywords:** Next generation sequencing, Error correction

## Abstract

**Background:**

Continued advances in next generation short-read sequencing technologies are increasing throughput and read lengths, while driving down error rates. Taking advantage of the high coverage sampling used in many applications, several error correction algorithms have been developed to improve data quality further. However, correcting errors in high coverage sequence data requires significant computing resources.

**Methods:**

We propose a different approach to handle erroneous sequence data. Presently, error rates of high-throughput platforms such as the Illumina HiSeq are within 1%. Moreover, the errors are not uniformly distributed in all reads, and a large percentage of reads are indeed error-free. Ability to predict such perfect reads can significantly impact the run-time complexity of applications. We present a simple and fast k-spectrum analysis based method to identify error-free reads. The filtration process to identify and weed out erroneous reads can be customized at several levels of stringency depending upon the downstream application need.

**Results:**

Our experiments show that if around 80% of the reads in a dataset are perfect, then our method retains almost 99.9% of them with more than 90% precision rate. Though filtering out reads identified as erroneous by our method reduces the average coverage by about 7%, we found the remaining reads provide as uniform a coverage as the original dataset. We demonstrate the effectiveness of our approach on an example downstream application: we show that an error correction algorithm, Reptile, which rely on collectively analyzing the reads in a dataset to identify and correct erroneous bases, instead use reads predicted to be perfect by our method to correct the other reads, the overall accuracy improves further by up to 10%.

**Conclusions:**

Thanks to the continuous technological improvements, the coverage and accuracy of reads from dominant sequencing platforms have now reached an extent where we can envision just filtering out reads with errors, thus making error correction less important. Our algorithm is a first attempt to propose and demonstrate this new paradigm. Moreover, our demonstration is applicable to any error correction algorithm as a downstream application, this in turn gives a new class of error correcting algorithms as a by product.

## Background

High-throughput short read sequencing technologies have become the mainstay of genomic research. Critical attention is paid to read quality as it affects the quality and performance of sequencing applications. For example, read quality directly impacts accuracy in mapping to a reference genome. In de novo genome sequencing, apart from accuracy of the generated contigs, read quality affects contig lengths. Error-free reads can also improve algorithmic performance, as alignments can be replaced with much faster exact matching.

The focus of this work is applications of high-throughput sequencing in which a single genome is sampled at high coverage, such as resequencing and de novo sequencing. In these cases, the infrequent occurrence of errors in reads, and the apparent lack of affinity of errors to any fixed genomic location, provide a way to detect and correct erroneous bases in reads. If the reads covering a specific genomic position can be identified and properly positioned relative to their locations of genomic occurrence, this layout can be used to infer the true base by majority vote and correct the others. This works for a haploid genome, but can be extended to polyploid genomes to at least identify correct bases. Several error correction algorithms for haploid genomes have been developed, using k-spectrum [1-4], suffix trees [5-7], or multiple sequence alignments [8,9] to identify overlapping reads. For a detailed survey of error correction methods, see [10,11].

Most error correction methods are designed for Illumina sequencers, which are predominantly used. With rare exceptions, reads from these sequencers only contain substitution errors, leading to simpler algorithms based on Hamming distance instead of edit distance. High-end sequencers have error rates well within 1%, and a large percentage of reads are indeed claimed to be free of errors. Taking advantage of this, in this paper we propose a different approach: rather than base-level error correction, we seek to identify reads that are error-free (or perfect ). If such predictions can be made with high accuracy, it opens the door to simplifying algorithms for downstream applications, not to mention improvements in quality. In fact, we show that error correction algorithms themselves can be improved by using the perfect reads to correct others, instead of collectively using all the reads.

### Contributions

In this paper, we present a k-spectrum analysis based approach to filter out erroneous reads in a high-coverage Illumina dataset. We applied our algorithm to one HiSeq 2500 and five HiSeq 2000 datasets. Our experiments show that if around 80% of the reads in a dataset are perfect, then our approach retains almost 99.9% of the perfect reads with more than 90% precision rate. Though the coverage reduces by 7% on an average, we observed no noticeable skew in the distribution of perfect reads as compared to the distribution of the original dataset. We also developed a way to characterize the type of datasets for which such an approach is effective.

Depending on the application, our method can be customized to vary the degree of stringency used to discard a read as erroneous. For example, if the objective is to retain most of the perfect reads despite the risk of increasing false positives, then the lowest level of stringency should be used. On the other hand, if the objective is to minimize false positives, the highest level of stringency should be used.

Finally, we demonstrate that our prediction of perfect reads can be used to improve the performance of error correction algorithms. To do so, we consider Reptile [1], a *k*-spectrum based error correction algorithm. This method performs an analysis of *k*mers in the input reads and uses a Hamming graph constructed based on the *k*mers to detect and correct errors. We found that if only *k*mers from perfect reads are used instead, this leads to an improvement of up to 10% on the percentage of errors removed from the dataset. This approach can be readily applied to improve other error correction algorithms.

The organization of the rest of the paper is as follows. The details of our approach are presented in Section titled Methods. Experimental results are presented in Section titled Results. In Section titled Improving error correction algorithms, we show how to apply this approach to improve error correction methods. We conclude in Section.

## Methods

Our algorithm is based on analyzing the k-spectrum of the given data set. The k-spectrum is the collection of all *k*mers, *i.e*., all substrings of length *k *from the reads. Define a *k*mer to be valid if it is present in the genome being sequenced, and invalid otherwise. A read is perfect if it does not contain any invalid *k*mer. In the absence of the reference genome, the validity of a *k*mer can be estimated from its frequency in the dataset. As errors are infrequent, with sufficient coverage, a valid *k*mer should occur at significantly larger frequency than invalid *k*mers. Thus, similar to *k*-spectrum based error correction algorithms, our method consists of two phases. In the first phase, we generate frequency statistics of the *k*mers, and construct a graph to link *k*mers within short Hamming distance. In the second phase, each read is checked for potential errors using the statistics built in the first phase.

### *k*mer statistics generation

The *k*mer at position p of read r is denoted by *r*[*p : p + k *− 1]. We assume k to be a fixed even number so that *k*/2 is a whole number. To determine the validity of a kmer, we also consider the quality scores of the associated bases. We count an instance of a kmer only if each of its associated bases exceeds a quality threshold *Q*_*E *_(stands for Excellent Quality). The number of such instances of a *k*mer *T *is termed its frequency, denoted by *f*(*T*). Because of the double stranded nature of DNA, each *k*mer is associated with its reverse complement *k*mer also. We represent both these *k*mers by the one smaller in lexicographic order, and combine the frequencies. The frequencies of all the *k*mers in the *k*-spectrum can be easily computed in a single pass over the data. Alternatively, other efficient *k*mer counters such as [12] can be modified to use quality scores.

In the first phase, our algorithm also builds a Hamming graph over the *k*-spectrum. Each *k*mer is represented by a node in the graph. A pair of nodes is connected if the corresponding *k*mers differ in at most d positions, for a fixed *d*. The main purpose of the graph is to better estimate the validity of a *k*mer by taking its graph neighborhood into account. We use the same space efficient data structure to construct and store the Hamming graph as in Reptile (for details, see [1]).

### Identifying perfect reads

Our algorithm for processing a read is presented in Algorithm 1. The algorithm decomposes a read into overlapping *k*mers such that the overlap between two consecutive *k*mers is k/2, half their length. If there are insufficient base pairs for such an overlap towards the end of a read, the last *k*mer is chosen to be the suffix of the read of length k. If none of these *k*mers is estimated to be invalid, the algorithm outputs the read as perfect.

Algorithm 1: *Error detection*

Data: Read *r*

Result: Classify *r *as Perfect or Erroneous

*p ← *0; /* current *k*mer *r*[*p:p+k*−1] */

while (*true*) do

*T ← k*mer of *r *at position p;

if *T *is not valid then return Erroneous;

if (*p + k = |r|*) then return Perfect;

if ((*p + k/*2*) + k < |r|*) then *p ← p + k*/2;

else *p ← |r| − k*;

end

The algorithm relies on a rule to estimate if a kmer is valid. We consider five different rules based on properties P1, . . . , P5 below:

P1: *f *(*T*) ≥ *C_E_*

P2: *f *(*T*) ≥ *C_G _*and each base pair in T has quality *≥ Q_G_*

P3: *f *(*T*) ≥ *C_G _*and T does not have a neighbor *T*′ in Hamming graph with *f*(*T*′) ≥ *C_G_*

P4: *f *(*T*) ≥ *C_G _*and T does not have a neighbor *T*′ in Hamming graph with *f*(*T*′) ≥ *f*(*T*) × *F_H_*

P5: *f *(*T*) ≥ *C_G _*and all neighbors *T*′ of *T *in the Hamming graph have property: all the base pairs of *T*

where *T *differs from *T*′ have quality score ≥ *Q*_*G *_where the following parameters are to be set appropriately: *C*_*E *_(Excellent Count ), *C_G _*(Good Count ), *Q_G _*(Good Quality), *F_H _*(High-cardinality Factor ), *C_E _> C_G _*and *Q_E _> Q_G_*.

For the rest of the paper, we say *T *is valid by Rule *i *if it satisfies any one of the properties P1, P2, . . . , P*i*. Thus, the rules are in decreasing order of stringency. In the most stringent case (P1), the algorithm treats a *k*mer *T *as valid only if its frequency *f* (*T*) is at least a threshold *C_E _*. In P2, *f* (*T*) is allowed to be above a lower threshold C_G _but each base in *T *must have quality score above *Q_G_*. The rationale for properties P3 and P4 is that as the *k*mer in consideration has comparatively lower frequency, and there are no high cardinality *d*-neighbors, it might be the case that the *k*mer is from a region of low coverage. In P5, the *k*mer has strong quality scores at all the positions in which it differs from its *d*-neighbors, and hence it has a high likelihood of being valid.

Note that these rules are heuristics and hence the perfect reads detected by our algorithm may have errors and some of the erroneous reads detected by our algorithm can in fact be error-free.

## Results

We applied our algorithm to 6 datasets from the NCBI short read archive, the details of which are given in Table 1. For each dataset the table shows SRA accession number, sequencer platform, name of the reference genome, strain, organization that published the data, date of publishing, percentage of *GC *content, length of the genome in Mb, read length, and average coverage.

**Table 1:** Sequence datasets.

Data Set	SRA Accession	Sequencing Platform	Reference Genome	Reference Strain	Organization	Publication Date	GC%	Genome Length (Mb)	Read Length	Coverage
S1	SRR789669	HiSeq 2500	D. Miranda	MSH22	UCB	08-05-2013	45.6	136.73	90	43
S2	SRR647546	HiSeq 2000	E. Coli	O157:H7	UMIGS	10-01-2013	51.7	5.59	101	369
S3	ERR036168	HiSeq 2000	P. Falciparum	3D7	WTSI	03-11-2011	22.5	23.27	75	362
S4	ERR142615	HiSeq 2000	B. Pertussis	ST24	WTSI	28-08-2012	67.2	4.12	75	1000
S5	ERR142617	HiSeq 2000	P. Falciparum	3D7	WTSI	28-08-2012	20.2	23.27	75	160
S6	SRR507777	HiSeq 2000	S. Cerevisiae	S288c	CSHL	20-06-2012	39	12.16	76	362

UCB: University of California, Berkeley; WTSI: Wellcome Trust Sanger Institute; UMIGS: Institute for Genome Sciences, University of Maryland; CSHL: Cold Spring Harbor Laboratory

To evaluate our method, knowledge of error-free reads in each dataset is required. To determine them, we aligned each dataset using the BWA aligner [13] with default parameters. A read is taken to be error-free if it is perfectly aligned by BWA without any substitution, insertion, or deletion. The results of BWA alignments are shown in Table 2 where each row shows the strain of the reference genome used, number of reads in the dataset, number of reads aligned, number of reads not aligned, number of reads ambiguously aligned, number of reads perfectly aligned, and overall error rate. Note that the rows of Tables 1 and 2 are arranged in increasing order of the percentage of perfect reads.

**Table 2:** Alignment of sequence datasets.

Data Set	Reference Strain	Number of Reads	Aligned (%)	Unaligned (%)	Ambiguous (%)	Perfect Reads (%)	Error Rate
S1	DroMir2.2	65603904	43580948 (66.4)	18010469 (27.5)	4012487 ( 6.1)	33234182 (50.7)	0.71
S2	O157:H7	20461442	18191534 (88.9)	1045418 ( 5.1)	1224490( 6.0)	13294580 (65.0)	0.72
S3	3D7	112418270	88192289(78.5)	19516044 (17.4)	4709936 ( 4.2)	74845636 (66.6)	0.43
S4	CS	54996906	46950786 (85.4)	3957391 ( 7.2)	4088729 ( 7.4)	44437437 (80.8)	0.24
S5	3D7	49738806	41836006 (84.1)	5818785 (11.7)	2084015 ( 4.2)	40472551 (81.4)	0.18
S6	S288c	57886340	46179933 (79.8)	2213383 ( 3.8)	9493023 (16.4)	51612399 (89.2)	0.13

### Experiments and evaluation methodology

We applied our algorithm to each of the datasets using the following default parameters: *k *= 24, *C_G _*= 1, *C_E _*= 8, *Q_G _*= 45. We chose *Q_E _*= 71 for S3 and *Q_E _*= 73 for the remaining datasets. To assess the quality of predictions made, we define:

*TP *= number of perfect reads that are classified by our algorithm as perfect

*FN *= number of perfect reads that are classified as erroneous

*FP *= number of erroneous reads that are classified as perfect

*TN *= number of erroneous reads that are classified as erroneous

Then, we used the standard measures of specificity (*S_p_*), sensitivity (*S_n_*), and precision (*P*_r_) as:

*S_p _*= *TN*/(*TN* + *FP*)

*S_n _*= *TP*/(*TP* + *FN*)

*P_r _*= *TP*/(*TP* + *FP*)

## Discussion

The results of our experiments using Rule 2, which tests for conformance with at least one of properties P1 and P2, are presented in Table 3. Except for dataset S2, Rule 2 achieves near 100% sensitivity, indicating this rule correctly classifies an overwhelming majority of error-free reads, and misclassifies a negligible percentage of error-free reads. Thus, applications which take reads predicted to be error-free by our algorithm will retain almost all of the error-free reads. Specificity for various datasets indicates to what extent our algorithm succeeded in weeding out reads that contain at least one error. Except for dataset S1, our algorithm eliminated at least 50% of erroneous reads from the dataset, reaching close to 90% in some cases (datasets S2 and S3). Precision, the ratio of true perfect reads to total reads predicted as perfect by our algorithm, is over 90% in all cases except dataset S1. The lower performance on S1 can be attributed to the comparatively lower coverage and lower percentage of perfect reads.

**Table 3:** Results using default parameters and Rule2.

Data Set	*TP*	*FN*	*FP*	*TN*	Precision *P_r_*	Specificity *S_p _*	Sensitivity *S_n _*	Time taken in seconds	
								**Phase 1 **	**Phase 2**

S1	3.3E+07	786	2.6E+07	6588766	0.563	0.204	1	149.786	217.319
S2	1.1E+07	2403877	781985	6384877	0.933	0.891	0.819	31.449	28.394
S3	7.5E+07	146338	4348595	3.3E+07	0.945	0.884	0.998	88.842	197.219
S4	4.4E+07	45171	3873795	6685674	0.92	0.633	0.999	51.391	148.213
S5	4E+07	51679	4422332	4843923	0.901	0.523	0.999	51.942	135.711
S6	5.2E+07	33420	1681374	4592567	0.968	0.732	0.999	54.857	160.928

**Figure 1:**
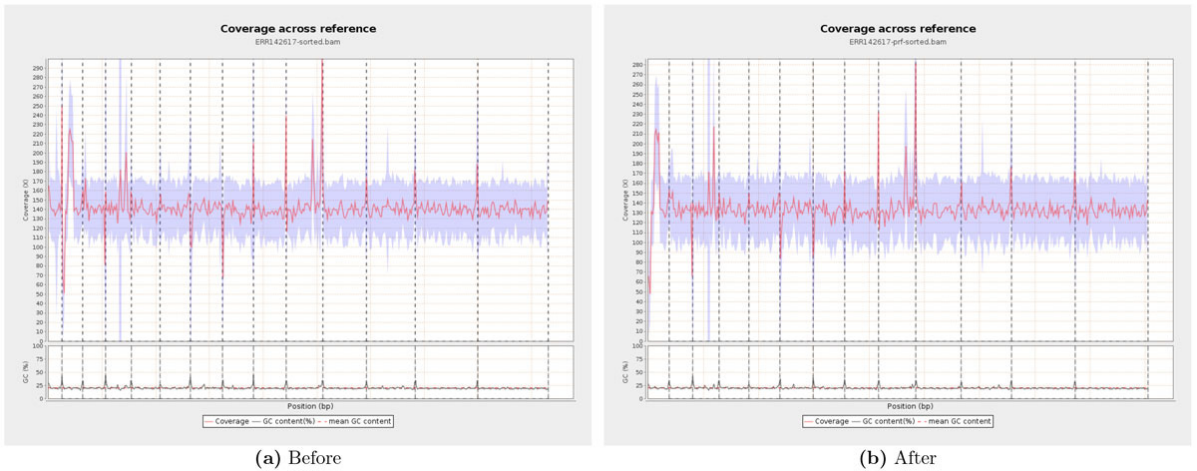
**Effect on coverage in dataset S5**.

In all cases except S1, applications can significantly benefit by taking as input the reads predicted to be error-free by our algorithm, instead of the raw datasets. Doing so, the applications will be operating on data that has over 90% perfect reads, miss very few perfect reads from the original dataset, and can do away with a majority of erroneous reads. We also tested the coverage induced by the reduced datasets generated by our algorithm against the coverage of the genome by the original raw datasets, and found no noticeable loss of information, i.e., we did not find any regions of the genome disproportionately losing coverage significantly higher than what is implied by the reduction in the size of the dataset. For visualization of the test, we show plots generated by the tool Qualimap [14], which divides the complete genome into about 400 windows and plots the average of the coverage of all base pairs within each window. We show in Figure 1 the plot generated by Qualimap for dataset S5 alone; for datasets S4 and S6 we get similar plots. It can be seen that the coverage pattern remains the same though the average coverage reduces from 140x to 130x (around 7%). Figure 1 also shows that the percentage of GC content remains same. As Illumina sequencers can generate billions of reads in a single experiment at a very low cost per base, eliminating erroneous sequences can significantly improve data quality for applications without appreciable loss of data-scale.

### Execution time

All experiments were carried out on a HP ProLiant DL580G7 server, which has 32 Intel Xeon E7520 (1.8 GHz) processors and 132GB main memory. The server is running 64-bit Ubuntu 12.04 OS. We used Jellyfish software [12] to generate *k*mer statistics in phase 1 of our algorithm. Our multi-threaded implementation of phase 2 is also based on the library functions associated with the Jellyfish software. The rightmost two columns of Table 3 show the average time taken by the two phases of our algorithm in 10 independent runs on each of our datasets. In our experimentation, we used 32 threads in each phase. The time taken in phase 2 is within the same order of magnitude of time taken in generating the *k*mer statistics.

### Predicting applicability of our algorithm

As noted previously, our algorithm performed well on all datasets except for S1. It would be of tremendous practical value if we can ascertain the applicability of our algorithm by evaluating the dataset alone, without knowing the reference genome. Below, we present a methodology to do so.

**Figure 2:**
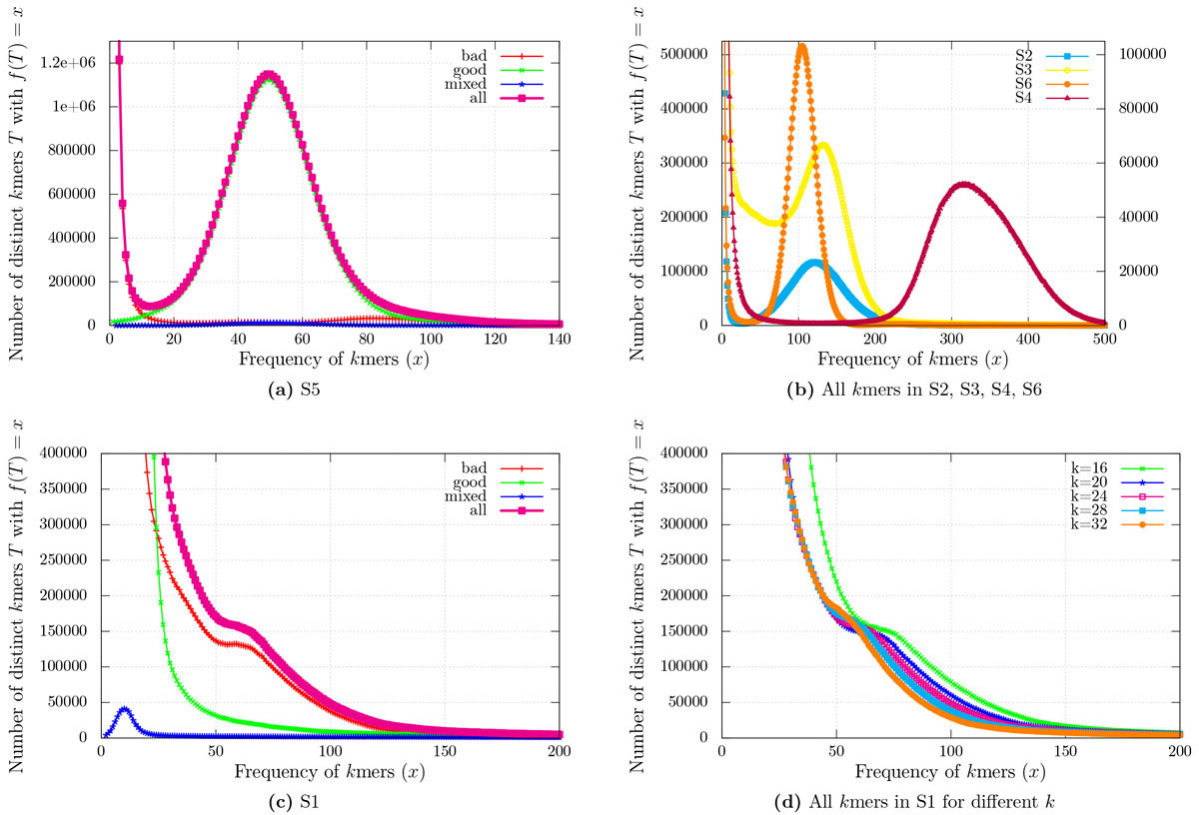
***k*mer frequency histogram**.

The quality of results obtained by our algorithm can be explained using the histogram of *k*mer frequencies (see Figure 2). In Figure 2(a) we plot the histogram of *k*mer frequencies in dataset S5 for *k *= 24. On the horizontal axis we show the different frequencies of the *k*mers. For a particular frequency *x *on the horizontal axis the curve named all shows the number of distinct *k*mers T that have frequency *f*(*T*) = *x*. Depending on the alignment, the *k*mers can be divided into three categories. A *k*mer is good if it comes from the error-free regions of all the reads that it appears, bad if it comes from erroneous regions in all the reads it appears, and mixed if it appears in the error-free regions on some reads and in the erroneous regions of some other reads. In Figure 2(a) we also show the frequency histograms for good, bad and mixed *k*mers.

A necessary condition for the good performance of a k-spectrum based error detection algorithm is that the frequency histograms for good, bad, and mixed *k*mers should follow the pattern in Figure 2(a) where the number of mixed *k*mers is very very low, most bad *k*mers have low frequency, bad *k*mers with higher frequencies are very rare, and the histogram of good *k*mer frequencies follows a normal curve which has mean approximately equal to the average coverage. When this condition is satisfied, we can clearly demarcate a threshold frequency at the crossover of the good and bad curves such that most of the *k*mers with frequency less than the threshold are bad, and most of the *k*mers with frequency above the threshold are good. The dataset S5 follows the pattern and our algorithm works well on S5.

Figure 2(b) shows the frequency histograms for all *k*mers in datasets S2, S3, S4, and S6, respectively. The plots follow the expected patterns, which explains the good performance of our algorithm on these datasets too. Note that S4 has comparatively higher coverage, and hence we used the additional right vertical axis with a different unit value.

On the other hand, the plots for S1 shown in Figure 2(c) do not follow the expected pattern. In fact it is not possible to distinguish the good *k*mers from the bad ones depending upon only the frequency, as the bad curve is above the good curve. Increasing the *k*mer size does not help either. Figure 2(d) shows the plots for all *k*mers for values of *k *= 16, 20, 24, 28, and 32, none of which follows the expected pattern. This explains the bad performance of our algorithm on S1.

Unlike the curves good, bad, and mixed, the curve all can be plotted even in the absence of alignment information. If the shape of the all curve follows the expected pattern as in Figure 2(d), our algorithm should perform well.

The plots also give hints on how to set the parameters. The parameter k should be such that the probability of a *k*mer having repeats in the genome *G, i.e*., |*G*|/4*^k ^*is very small. C_E _should be the first minimum of all curve. At least 95% of the *k*mers should have frequency C_G _or more. The quality parameters could be such that about 80% (20%) of the bases have quality at least *Q_G _*(*Q_E _*).

### Effects of varying stringency levels

We also varied the Rules used to identify if a *k*mer is valid or not, as described in Section. We report the results on datasets S4, S5 and S6 with parameter *F_H _*= 2 in Table 4. As we increase the rule number, the stringency of declaring a read to be error-free decreases, resulting in more true and false positives. Hence, sensitivity increases but specificity and precision decrease.

**Table 4:** Results on S4, S5, and S6 for varying rules.

Data Set	Rule	*TP*	*FN*	*FP*	*TN*	*P_r_*	*S_p_*	*S_n_*
S4	Rule1	44315371	122066	3718959	6840510	0.923	0.648	0.997
S4	Rule2	44392266	45171	3873795	6685674	0.920	0.633	0.999
S4	Rule3	44392266	45171	3873932	6685537	0.920	0.633	0.999
S4	Rule4	44415654	21783	3905218	6654251	0.919	0.630	1.000
S4	Rule5	44416777	20660	3926550	6632919	0.919	0.628	1.000

S5	Rule1	40246703	225848	4161367	5104888	0.906	0.551	0.994
S5	Rule2	40420872	51679	4422332	4843923	0.901	0.523	0.999
S5	Rule3	40420999	51552	4422972	4843283	0.901	0.523	0.999
S5	Rule4	40433782	38769	4435544	4830711	0.901	0.521	0.999
S5	Rule5	40443582	28969	4442097	4824158	0.901	0.521	0.999

S6	Rule1	51341705	270694	1354288	4919653	0.974	0.784	0.995
S6	Rule2	51578979	33420	1681374	4592567	0.968	0.732	0.999
S6	Rule3	51579033	33366	1681628	4592313	0.968	0.732	0.999
S6	Rule4	51605360	7039	1684518	4589423	0.968	0.732	1.000
S6	Rule5	51605719	6680	1690727	4583214	0.968	0.731	1.000

## Improving error correction algorithms

Our algorithm for predicting error-free reads can be used to improve the performance of error correction algorithms themselves. We demonstrate this using the error correction algorithm Reptile [1], though the methodology is more broadly applicable. Reptile consists of two phases: In the first phase, it counts the frequency of all *k*mers and constructs a Hamming graph on them. In addition, *k*mers are classified as valid or invalid based on whether or not they exceed a threshold count. Nodes in the Hamming graph are marked with this information. In the second phase, each read is corrected by changing invalid *k*mers in it to their valid Hamming graph neighbors. We slightly modified the first phase of Reptile to count *k*mer frequencies in only those reads which are predicted as error-free by our algorithm. In addition, we use the Hamming graph to only correct errors in reads that are predicted to be erroneous by our algorithm.

Table 5 compares the performance of the modified Reptile algorithm with the original Reptile algorithm. For each dataset, we show performance of the original Reptile algorithm, followed by the modified algorithm using Rule 1 and Rule 2, respectively. For comparison, we use the measures described in [1]: a *TP *is any erroneous base that is changed to true base, an *FP *is any true base changed wrongly, a *TN *is any true base left unchanged and an *FN *is any erroneous base left unchanged. We also report Gain *G *= (*TP *− *FP*)/(*TP* + *FN*), which measures the percentage of errors removed from the dataset, and *EBA *= *WC*/(*TP* + *WC*) where *WC *denotes the number of erroneous bases that are correctly identified but changed to a wrong base.

**Table 5:** Results on reptile error correction.

*Set*	*Algo*	*TP*	*FN*	*FP*	*TN*	*WC*	*S_n_*	*S_p_*	*G*	*EBA*
S1	Reptile	4780231	28675648	134010	4249799767	19494	0.143	1.000	0.139	0.004061
S1	Rule1	1299909	6308111	3427119	1143304758	18463	0.171	0.997	-0.280	0.014004
S1	Rule2	9147518	24304818	158446	4249775331	23037	0.273	1.000	0.269	0.002512

S2	Reptile	5665375	8695457	90366	1946564438	2788	0.395	1.000	0.388	0.000492
S2	Rule1	2895901	11462701	745833	1945908971	5018	0.202	1.000	0.150	0.001730
S2	Rule2	5371516	8988883	128454	1946526350	3221	0.374	1.000	0.365	0.000599

S3	Reptile	18025179	10286737	605575	6874673712	44797	0.637	1.000	0.615	0.002479
S3	Rule1	18806026	9498842	1940111	6873336908	51638	0.664	1.000	0.596	0.002738
S3	Rule2	18290440	10019429	695540	6874583747	46844	0.646	1.000	0.622	0.002555

S4	Reptile	5565750	3453627	17740	3810574694	5139	0.617	1.000	0.615	0.000922
S4	Rule1	6365587	2653084	50006	3810542428	5845	0.706	1.000	0.700	0.000917
S4	Rule2	5990892	3028178	19705	3810572729	5446	0.664	1.000	0.662	0.000908

S5	Reptile	1690826	3501370	302635	3259337078	11016	0.326	1.000	0.267	0.006473
S5	Rule1	1904825	3286174	481440	3259158273	12213	0.367	1.000	0.274	0.006371
S5	Rule2	1802536	3389315	341530	3259298183	11361	0.347	1.000	0.281	0.006263

S6	Reptile	4346955	1083849	16099	4213859720	1413	0.800	1.000	0.797	0.000325
S6	Rule1	4939773	490989	37325	4213838494	1455	0.910	1.000	0.903	0.000294
S6	Rule2	4449982	980530	19259	4213856560	1705	0.819	1.000	0.816	0.000383

As proposed in [1] and more widely adopted later, Gain is an important measure for assessing the quality of an error correction algorithm. From Table 5 for the datasets on which our prediction performs well, mainly S4, S5, and S6, the modification improves Gain by up to 10%.

## Conclusions

Thanks to the continuous technological improvements in high-throughput DNA sequencing, reads of dominant sequencing platforms such as the Illumina HiSeq are sporting high coverage and accuracy. This has now reached an extent where we can envision just filtering out reads with errors, thus making error correction less important. Our algorithm is a first attempt to propose and demonstrate this new paradigm. Our experimental results demonstrate that development of such algorithms shows great promise. There are directions for further improvement of our algorithmic strategy. Our algorithm relies on several parameters. An automated choice of parameters sensitive to, and computed based on, the dataset would be useful. It might be useful to have values of parameters *C_E _, C_G _*dependent on the pattern of bases in the *k*mers. Like all error correction algorithms, our algorithm ignores paired read information and treats them as though they are single reads. Utilizing paired read information to further improve the performance of error detection or correction algorithms remains an open question. In case of long reads where reads are less likely to be perfect, a notion of approximate perfect (say at most *e *errors) can be used.

## Availability

A C++ based implementation of our algorithm can be found at the following github public repository: https://github.com/soumitrakp/perfectread.git.

## Competing interests

The authors declare that they have no competing interests.

## Authors' contributions

SA conceived the study. SP implemented the algorithms and carried out the experiments. SP and SA analyzed the results and wrote the paper.

## Declarations

Publication of this article was funded by U.S. National Science Foundation awards ATD-1120597 and CCF-1360593.

This article has been published as part of *BMC Bioinformatics *Volume 16 Supplement 17, 2015: Selected articles from the Fourth IEEE International Conference on Computational Advances in Bio and medical Sciences (ICCABS 2014): Bioinformatics. The full contents of the supplement are available online at http://www.biomedcentral.com/bmcbioinformatics/supplements/16/S17.
